# 

**Published:** 1959-07

**Authors:** 


### *Contents*

DECAY OF THE TEETH . . . . . *A. I. Darling* 53

LUNG CANCER—A SYMPOSIUM . . . *Dr. J. E. G. Pearson* 61

ALVEOLAR CELL CARCINOMA OF LUNG 70
*Clinical Pathological Conference*

NEWS FROM SOCIETIES . . . . . . . . 74

NOTES AND NEWS . . . . . . . 77

APPOINTMENTS . . . . . . . . 78

